# Phylogeny and Taxonomy of the *Naematelia aurantialba* Complex in Southwestern China

**DOI:** 10.3390/jof10120845

**Published:** 2024-12-06

**Authors:** Jin-Yan Tang, Zhu-Liang Yang

**Affiliations:** 1 Key Laboratory of Phytochemistry and Natural Medicines, Kunming Institute of Botany, Chinese Academy of Sciences, Kunming 650201, China; tangjinyan@mail.kib.ac.cn; 2 Yunnan Key Laboratory for Fungal Diversity and Green Development, Kunming 650201, China; 3 University of Chinese Academy of Sciences, Beijing 100049, China

**Keywords:** genome skimming approach, Naemateliaceae, phylogeny, taxonomy, *Tremellales*

## Abstract

*Naematelia aurantialba* and its allies are important edible and medicinal mushrooms in China. They are usually called Jiner (金耳) and have been cultivated on a commercial scale. However, due to the lack of DNA sequences from the holotype of *Naematelia aurantialba*, the taxonomic issues of the species complex are unresolved. In this study, the authors successfully generated DNA sequences from the holotype of *N. aurantialba* by a genome skimming approach and additional allied species by Sanger sequencing. Based on morphological characteristics, molecular phylogenetic data, and geographic distribution patterns, four species, including three new ones, in the complex in southwestern China were uncovered. *Naematelia aurantialba* occurs at high altitudes (over 3000 m above sea level), with subalpine dead plants as its substrates, and has larger basidiospores, while the commonly cultivated species, described as *N. sinensis* in this work, is distributed in subtropical areas at altitudes between 1800 m and 2600 m on the dead wood of subtropical plants and has smaller basidiospores. The third species, namely *N. nodulosa*, has habitats similar to those of *N. sinensis* but differs from the latter in its basidiomata with an uneven nodulose surface, a loose context with small internal cavities, and numerous conidia. The fourth species, *N. pedicellata*, is easily distinguished from the others by its basidia, with long basal stalks and broadly ellipsoid basidiospores measuring 10.5–12.5 × 8.0–10.0 μm. All these species are parasitic on *Stereum* species. This study provides a solid basis for future guidance for the selection of new strains and cultivation practices of these valuable fungi.

## 1. Introduction

*Naematelia* Fr. is one of the two genera in the family Naemateliaceae, order *Tremellales* [1]. This genus was introduced by Fries [2] for fungi having basidiomata with a firm, gelatinous outer layer and a hard inner core. Clements and Shear [3] designated *N. encephala* (Pers.) Fr. as the type of the genus [4]. Some mycologists subsequently used the name, while others considered *Naematelia* as a synonym of *Tremella* Pers. [5,6]. However, molecular phylogenetic analyses have indicated that *Naematelia* and *Tremella* represent unrelated phylogenetic lineages [7,8,9,10,11], and thus, the name *Naematelia* was resurrected by Liu et al. to accommodate the species complex of *N. encephala-N. aurantia* (Schwein.) Burt [1]. Over 10 species are described in *Naematelia* (Index Fungorum https://www.indexfungorum.org, accessed on 28 October 2024), and 5 of them, namely *N. aurantia*, *N. microspora*, *N. aurantialba*, *N. encephala*, and *N. encephaloidea*, are better known than the others due to the additional reports and availability of DNA sequences of these 5 species. It is known that *N. aurantia* and *N. encephala* have a broader distribution range than *N. aurantialba* and *N. encephaloidea* [6,7,11,12,13,14,15,16,17].

The basidiomata of the genus are cerebriform, tuberculate or spherical, or thick foliaceous with folds and ridges; lobed to frondose; pinkish, ochraceous, yellow, or brown; firm, gelatinous, and compact, with the inner part whitish and hard. Microscopically, basidiomata produce clamped hyphae and have haustorial cells from which hyphal filaments attach to and penetrate the unclamped hyphae of the host. The basidia are tremelloid, globose to ellipsoid, and longitudinally or obliquely septate, with probasidia and epibasidia on which basidiospores are produced. Basidiospores are smooth and globose to ellipsoid and germinate by budding and/or by repetition. Conidiophores are often present and produce conidia [1,7,13,14].

All species of *Naematelia* are parasitic on the basidiomata of *Stereum* species, including *S. hirsutum* (Willd.) Fr. on broadleaf trees and *S. sanguinolentum* (Alb. & Schwein.) Fr. on conifers. A single micropore connects the cytoplasm of the haustorial filament with that of the host cell [15]. One species is commercially cultivated for food and traditional Chinese medicine in China and is usually called Jiner (金耳) “*Naematelia aurantialba*” in the literature [18,19]. This species is rich in various nutrients and has various antioxidant, anti-inflammatory, anti-tumor, and immune-regulatory properties and, thus, is an important edible and medicinal fungus in China [20,21,22,23,24]. However, with increasingly more phylogenetic data on Jiner, it has become evident that a species complex of fungi has been dealt with [12,25]. Because no DNA sequences from the holotype of *N. aurantialba* (Bandoni & M. Zang) Millanes & Wedin are available, the systematic position of *N. aurantialba* cannot be anchored, and the taxonomic issues of this species complex are unresolved.

The aim of our study was (1) to generate DNA sequences from the holotype of *N. aurantialba* and other materials collected from southwestern China and (2) to reveal and characterize the species diversity of the complex in southwestern China.

## 2. Materials and Methods

### 2.1. Morphological Characterization

In this study, a total of 20 specimens of the genus *Naematelia* were collected. The voucher specimens were photographed and dried in the field and then stored in the Herbarium of Cryptogams at the Kunming Institute of Botany, Chinese Academy of Sciences (KUN-HKAS). Macroscopic descriptions were based on field records and digital images of the basidiomata. Microscopic features were observed by soaking hand-cut sections of the tissues in 5% KOH (Guangdong Guanghua Sci-Tech Co., Ltd., Guangzhou, Guangdong, China) and staining with 1% Congo red (Tianjin Guangfu Technology Development Co., Ltd., Tianjin, China) solution when necessary [7]. In the description of basidiospores, the abbreviation (n/m/p) means n basidiospores measured from m basidiomata of p collections. Dimensions for basidiospores are given using a range notation of the form (a–) b–c (–d). The range b–c contains a minimum of 90% of the measured values. Extreme values a or d are given in parentheses. Q represents the length–width ratio of a basidiospore in side view. Qm means the average Q of all basidiospores measured ± the sample standard deviation.

### 2.2. DNA Extraction, Sequencing, and Data Processing

**Figure 1:**
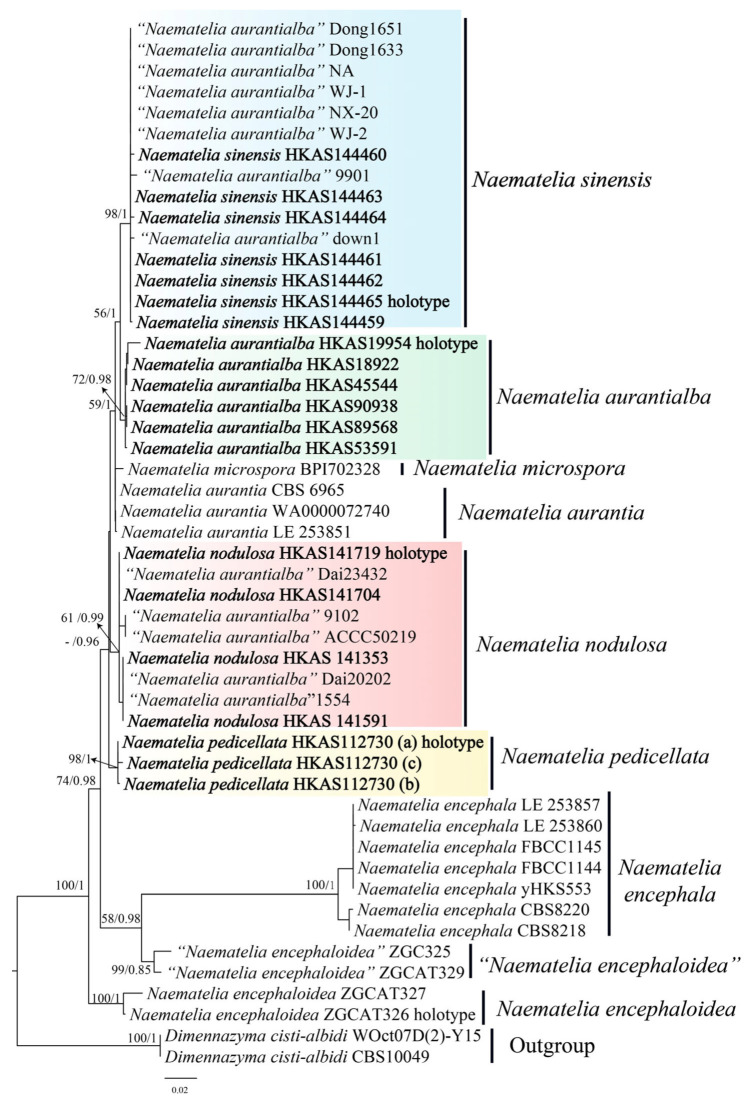
Phylogenetic tree of *Naematelia* with related species based on maximum likelihood analysis from a two-loci (ITS, nrLSU) dataset. ML ≥ 50% and BI ≥ 70% are indicated above the branches.

This study used Sanger sequencing and next-generation sequencing. In the Sanger sequencing process, the experimental procedures for DNA extraction, PCR amplification, and sequencing followed the descriptions provided by Cai et al. [26] and their cited references. Due to the limited number of sequences for *Naematelia* in the NCBI and the availability of only ITS and nrLSU sequences for the type specimen of *N. aurantialba*, this study used only ITS and the nrLSU to construct a molecular phylogenetic tree (Figure 1). The sequencing data were examined and aligned using SnapGene (GSL Biotech LLC, Boston, MA, USA) to check the peak plots of the sequencing results, removing low-quality regions. A total of 36 sequences were obtained and submitted to GenBank.

Second-generation sequencing of the type specimen of *Naematelia aurantialba* (HKAS19954, HKAS18922) was performed on the Illumina HiSeq platform. Protocols for DNA extraction, library preparation, and library pooling for genome sequencing followed those described in Zeng et al. [27] for genome skimming. Genome skimming is an efficient sequencing method that involves shallow sequencing of the genome. Compared to whole-genome sequencing, genome skimming has lower costs and generates smaller data volumes, but it allows for the rapid acquisition of key species information, such as species identification and phylogenetic analysis. Having obtained second-generation genomic data, we performed quality control using fastp v0.23.2 (Shenzhen Institutes of Advanced Technology, Chinese Academy of Sciences, Shenzhen, China) [28], which involved removing low-quality sequences, eliminating base contaminants, and performing correction. After Fastp data quality control, genome assembly was conducted using SPAdes genome assembler v3.15.5 (Illumina Hayward, Hayward, CA, USA) [29]. Afterward, library comparisons were performed, and two target fragments, namely internal transcribed spacers (ITS) and the nuclear ribosomal large subunit (nrLSU), were extracted from the assembled genome contigs.fasta. The obtained sequences were then subjected to comparison using the NCBI’s BLAST to assess their reliability. A total of 4 sequences were obtained using this method.

### 2.3. Phylogenetic Analysis

The DNA sequences generated in this study were aligned with those obtained from GenBank (Table 1) using MAFFT v7.490 (Biomatters, New Zealand, Auckland) [30] with the L-INS-i strategy for accurate alignment. The aligned results were then converted from text format to FASTA format using Geneious Prime v 2023.2.1 (Biomatters, Auckland, New Zealand). Subsequently, matrix adjustment was performed using PhyDE 0.9971 (Muenster, Germany), and the adjusted DNA sequences were used with RAxML v8.2.12 (Heidelberg Institute for Theoretical Studies, Heidelberg, Germany) [31] to construct a phylogenetic tree. The substitution model was set to GTRGAMMAI, and bootstrap values were obtained through 1000 repetitions of nonparametric bootstrapping. Bayesian inference (BI) analysis uses the Concatenate Sequence tool in PhyloSuite v1.2.2 (Institute of Hydrobiology, Chinese Academy of Sciences, Wuhan, China) [32] to combine multiple sequences in a specified order, generating a new complete sequence. PartitionFinder v2.0 (Australian National University, Canberra, Australia) [33] was then used to determine optimal nucleotide substitution models for each data partition. The best model for both ITS and the LSU is TRNEF+G. A phylogenetic tree was constructed using MrBayes 3 (Department of Systematic Zoology, Evolutionary Biology Centre, Uppsala University, Uppsala, Sweden) [34] with the following parameter settings: number of MCMC chains, 4 chains (1 cold chain + 3 hot chains); generations, a total of 2,000,000 generations, with sampling every 1000 generations; initial burn-in 25% of the total samples discarded as burn-in data; chain convergence, convergence assessed by average standard deviation <0.01; and node support, posterior probability values from the MrBayes output used as node support (Figure 1).

### 2.4. Availability of Data and Materials

The sequence data obtained in this study have been uploaded to the NCBI (https://submit.ncbi.nlm.nih.gov/, accessed on 29 November 2024), and the phylogenetic-tree-related files have been submitted to TreeBASE (https://treebase.org/treebase-web/user/submissionList.html, accessed on 10 November 2024).

## 3. Results

### 3.1. Molecular Analyses

The phylogenetic trees inferred from the ML and BI analyses were similar in topology. Therefore, only the tree obtained from the ML analysis is presented (Figure 1). Our molecular phylogenetic inference based on the nucleotide sequences of ITS and the nrLSU suggested that four distinct phylogenetic lineages of *Naematelia* occur in southwestern China, and they are closely related to each other. Although the relationships among most of the lineages are not resolved, it is evident that collections of *Naematelia aurantia* are close to the type of *N. microspora* (Figure 1). Two collections, ZGC325 and ZGCAT329, regarded as “*N. encephaloidea*” are different from the true *N. encephaloidea,* containing the type (Figure 1). Examinations of the collections indicated that each lineage has its own unique morphological features and distribution ranges. Consequently, these lineages are regarded as independent species.

### 3.2. Taxonomy

**Figure 2:**
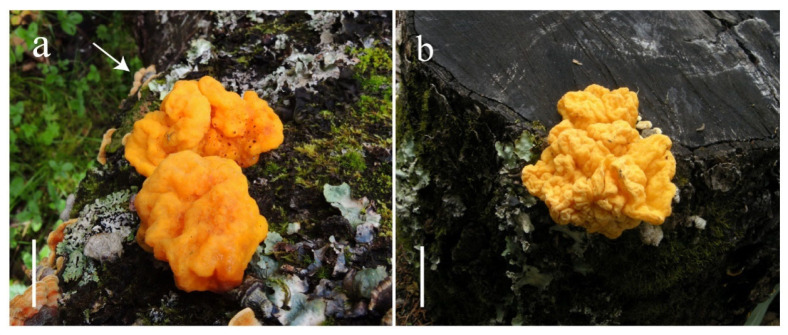
*Naematelia aurantialba*: (**a**,**b**) fresh basidiomata; note the *Stereum* basidiomata (arrow); (**a**) HKAS 89568; (**b**) HKAS 90938). Bars = 20 mm.

**Figure 3:**
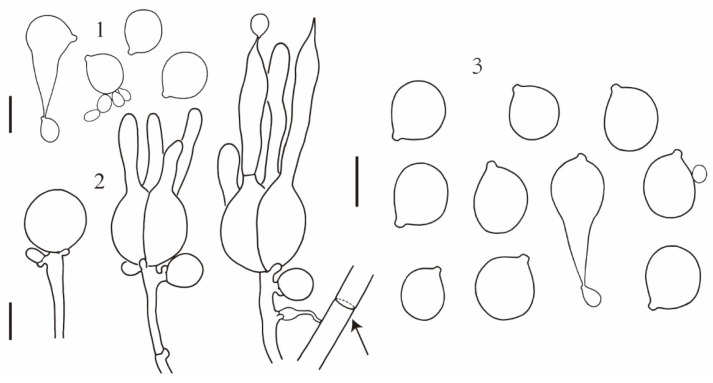
*Naematelia aurantialba*: (1) basidiospores germinating by budding or by repetition (HKAS89568); (2) basidia at different stages of development; a haustorium attached to a host hypha (arrow) (HKAS89568); (3) basidiospores germinating by budding or by repetition (HKAS19954, holotype). Bars = 10 μm.

***Naematelia aurantialba* (Bandoni & M. Zang) Millanes & Wedin. (Figure 2 and Figure 3)**.

**Type:** China, Sichuan Province, Kangding County, Liuba, alt. 3100 m, 24 July 1984, Ming-Sheng Yuan 369 (HKAS19954).

**Description:** Basidioma 1–4 cm high, 3.5–15 cm in diameter, subglobose, flattened hemispherical to lobed or irregular, bright yellow or lemon yellow to yellow brown, firm, gelatinous; surface wrinkled to contorted; flesh up to 4 cm thick, light yellow to dirty white, gelatinous, with or without cavities; brownish to yellow brown and horny when dried (Figure 2). Mature probasidia 18–22 × 15–20 μm, subglobose to ovoid, longitudinally cruciate-septate, four spored; epibasidia up to 100 μm long and 6 μm in diameter, nearly cylindrical to fusiform. Basidiospores [55/4/4] (8.5) 9.5–12.5 (13.0) × (8.0) 8.5–11.5 (12.0) μm, Q = (1.0)1.04–1.22(1.24), Qm = 1.11 ± 0.06, subglobose to broadly ellipsoid, smooth, germinating by budding or by repetition. Conidia 2–5 × 2–4 μm, globose, subglobose, ovoid to ellipsoid, thin walled, colorless, produced by basidiospores or by conidiogenous cells. Trama with parasite hyphae and host hyphae; parasite hyphae 2–5 μm wide, colorless, thin walled to slightly thick walled, with clamp connections; haustoria numerous, clamped, haustorial filaments rarely branched; host hyphae abundant in the inner part but also present up to the subhymenium of the parasite (Figure 3).

**Habitat and distribution:** Parasitizing species of *Stereum hirsutum* or its allies growing on decayed logs of subalpine *Quercus* species in the summer and autumn; known from Sichuan, Yunnan, and Xizang (Tibet).

**Additional collections examined:** China: Sichuan Province, Kangding County, Gongga Mountain Township, Liuba, 22 June 1984, Ming-Sheng Yuan 169 (HKAS18922); Sichuan Province, Derong County, Xiagajin Mountain, alt. 3600 m, 19 July 2004, Zhu-Liang Yang 4158 (HKAS45544); Sichuan Province, Garze Prefecture, Danba County, Dongma Village, 24 July 2007, Zai-Wei Ge 1505 (HKAS53591). Xizang (Tibet) Autonomous Region, Nyingchi City, Baxi Town, Niangdang Village, alt. 3812 m, 30 July 2014, Kuan Zhao 637 (HKAS89568); Nyingchi City, Baxi Town, 29 July 2014, Jian-Wei Liu 144 (HKAS90938).

**Notes:*** Naematelia aurantialba* has a yellow, nearly subglobose-to-hemispherical basidioma with a contorted surface. In comparison with other species of the species complex, the basidia and basidiospores are relatively larger, and conidia are produced both inside and on the surface of the basidioma but in relatively small quantities.

*Naematelia aurantialba* is close to *N. aurantia* in morphology and phylogeny (Figure 1). However, the latter species has a convoluted frondose basidioma with hollow lobes and subglobose-to-ellipsoid basidia, often with a distinct stalk, and broadly ellipsoid-to-ovoid basidiospores up to 10.5 μm long and 9 μm wide [5,7,11].

*N. aurantialba* also resembles *N. sinensis* but differs from the latter in its larger basidia and basidiospores and subalpine temperate distribution at altitudes above 3000 m in southwestern China. *Naematelia aurantialba* looks like *N. encephaloidea* A. Thomas & T.K.A. Kumar, originally described from Kerala, a state in India. However, the latter species has much smaller basidia, measuring 11–16 × 10–13 μm, and significantly smaller basidiospores, measuring 6.2–9.0 × 5.0–7.0 μm [12].

**Figure 4:**
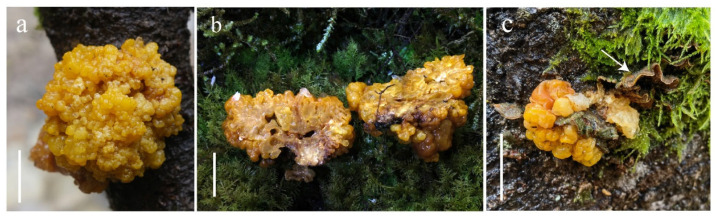
*Naematelia nodulosa* (HKAS141719, holotype): (**a**) fresh basidioma; (**b**) vertical sections of basidioma; (**c**) fresh basidioma and host, namely *Stereum hirsutum* or its allies (arrow). Bars = 20 mm.

**Figure 5:**
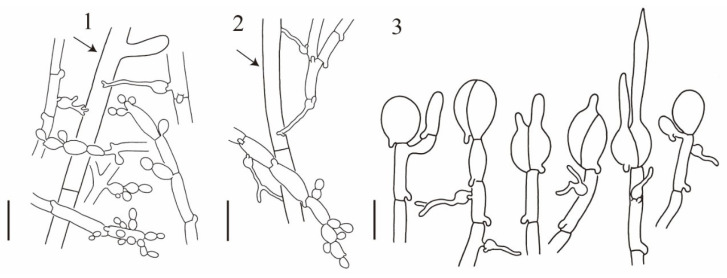
*Naematelia nodulosa*: (1) parasite hyphae with haustoria, conidiophores and conidia, and unclamped host hypha (arrow) in trama (HKAS 141591); (2) parasite hyphae with haustoria, conidiophore and conidia, and unclamped host hypha (arrow) in trama (HKAS141719, holotype); (3) basidia at different stages of development (HKAS141719, holotype). Bars = 10 m.

***Naematelia nodulosa* Zhu L. Yang & J.Y. Tang, sp. nov. (Figure 4 and Figure 5).**

**MycoBank:** MB 856508

**Etymology:** Named for the knob-like appearance of the surface of the basidioma.

**Type:** China, Yunnan Province, Lushui City, Pianma Town, alt. 1952 m, 12 August 2023, Xuan Chen GLG-CX170 (HKAS 141719, holotype).

**Diagnosis:** This species is similar to *N. sinensis* but differs from the latter in terms of its basidioma with a nodulose surface, a common presence of internal cavities, often two-spored smaller basidia, and numerous conidia.

**Description:** Basidioma 3–7 cm in diameter, globose to subglobose to hemispherical, yellow to golden yellow, firm and gelatinous; surface with an irregular profile, mostly nodular in shape; flesh up to 4 cm thick, dirty white to pale yellow, with small cavities; turning dark yellow to brown and becoming horny when dried (Figure 4). Mature probasidia 10–16 × 10–13 μm, subglobose, ovoid to broadly ellipsoid, longitudinally or diagonally septate, two spored, occasionally four spored; epibasidia up to 30 μm long and 4 μm in diameter, nearly cylindrical to fusiform. Basidiospores not observed. Conidia 2–6 × 2–4 μm, numerous, globose, subglobose, ovoid to ellipsoid, thin walled, colorless, and extensively distributed on the surface and in the trama of basidioma. Trama with parasite hyphae and host hyphae; parasite hyphae 2–5 μm wide, colorless, thin walled to slightly thick walled, with clamp connections; haustoria numerous, clamped, haustorial filaments, sometimes branched; host hyphae abundant in the inner part but also present up to the subhymenium of the parasite (Figure 5).

**Habitat and distribution:** Parasitizing species of *Stereum hirsutum* or its allies growing on decayed broadleaved tree trunks in the summer; known from western Yunnan.

**Additional collections examined:** China, Yunnan Province, Lushui City, Pianma, alt. 1950–2000 m, 12 August 2023, Jin-Yan Tang GLG-785 (HKAS 141591); Xue-Ping Fan GLG-FXP1592 (HKAS 141353); GLG-CX153 (HKAS 141704).

**Notes:** *Naematelia nodulosa* has a nearly spherical basidioma with a nodulose surface and internal cavities, small two-celled probasidia, and numerous conidia produced both in the trama and on the surface of the basidioma. Both *N. nodulosa* and *N. sinensis* can be found in subtropical forest areas. However, *N. nodulosa* differs from the latter in its basidiomata with a nodulose surface, a common presence of cavities in the trama, often two-spored smaller basidia, and numerous conidia.

**Figure 6:**
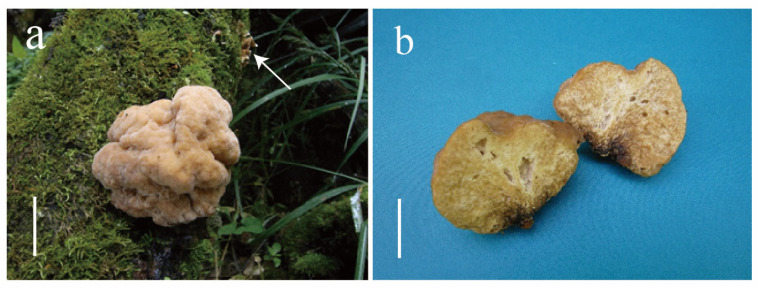
*Naematelia pedicellata* (HKAS 112730, holotype): (**a**) fresh basidioma; note the *Stereum* basidiomata on the upper right of the trunk (arrow); (**b**) vertical sections of basidioma. Bars = 20 mm.

**Figure 7:**
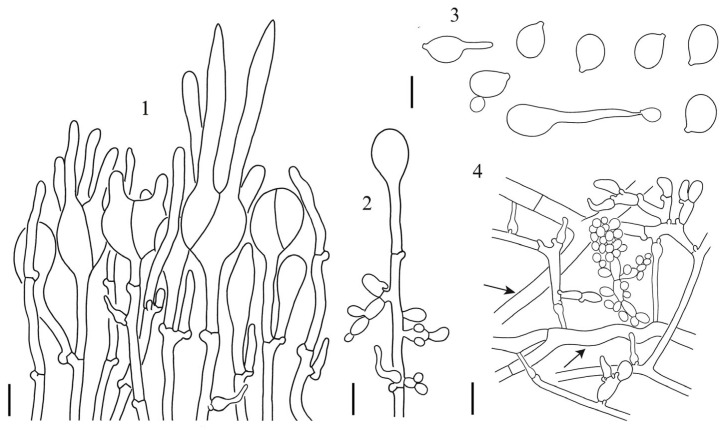
*Naematelia pedicellata* (HKAS 112730, holotype): 1. hymenium with stalked basidia at different stages of development and hyphidia; 2. cell immediately below a basidium with conidia; 3. basidiospores, germinating by budding or by repetition; 4. parasite hyphae with haustoria, conidiophore and conidia, and unclamped hyphae of host (arrows) in trama. Bars = 10 μm.

***Naematelia pedicellata* Zhu L. Yang & J.Y. Tang, sp. nov. (Figure 6 and Figure 7).**

**MycoBank:** MB 856509

**Etymology:** Named for the slender and elongated stalks of its basidia.

**Type:** China, Yunnan Province, Kunming City, near Miaogao Temple in Xishan Forest, alt. 2200 m, Zhu-Liang Yang 6334 (HKAS 112730, holotype).

**Diagnosis:** This species resembles *N. sinensis*, but it differs from the latter in its dull-colored basidiomata, long-stalked basidia, and elongated basidiospores.

**Description:** Basidioma 5 cm in diameter, nearly subglobose, dull ochraceous, firm and gelatinous; surface rugose, with irregular grooves; flesh up to 4 cm thick, dirty white to brownish, with small cavities; turning dark brown and becoming horny when dried (Figure 6). Mature probasidia 17–20 × 15–18 μm, subglobose to ovoid, usually diagonally septate and four spored, with long stalks (up to 40 μm); epibasidia up to 70 μm long and 5 μm in diameter, nearly cylindrical to nearly fusiform. Basidiospores [20/1/1] (10.0)10.5–12.5 (13.0) × 8–10 (11.0) μm, Q = (1.16)1.18–1.38(1.56), Qm = 1.25 ± 0.09, broadly ellipsoid, occasionally ellipsoid, smooth, germinating by budding or by repetition. Conidia 3–5 × 2–4 μm, abundant, globose, subglobose to ovoid, thin walled, colorless, produced by basidiospores or by conidiogenous cells, distributed both in the trama and on the surface of the basidioma. Trama with parasite hyphae and host hyphae; parasite hyphae 2–5 μm wide, colorless, thin walled to slightly thick walled, with clamp connections; haustoria numerous, clamped, haustorial filaments rarely branched (Figure 7).

**Habitat and distribution:** Parasitizing species of *Stereum hirsutum* or its allies growing on decayed wood in subtropical broadleaved forests in the summer; known from central Yunnan.

**Notes:*** Naematelia pedicellata* has dull colored basidioma with small cavities in the trama; 4 basidia with long, slender stalks at the base; relatively large basidiospores; and numerous conidia produced in the trama and on the surface of the basidioma. This species resembles *N. sinensis* but has a dull colored basidioma, long-stalked basidia, and basidiospores with a higher Q.

*Naematelia aurantia* also possesses stalked basidia. However, *N. aurantia* has foliaceous basidioma and significantly smaller basidiospores [5,7,11]. Weathered specimens of *N. encephala* (Pers.) Fr. may be confused with *N. pedicellata*. But, the former species has smaller subglobose basidiospores, measuring 9.0–11.0 × 7.5–9.0 μm [16,17], and its host is *Stereum sanguinolentum* (Alb. & Schwein.) Fr., which is usually on coniferous wood [13,17].

**Figure 8:**
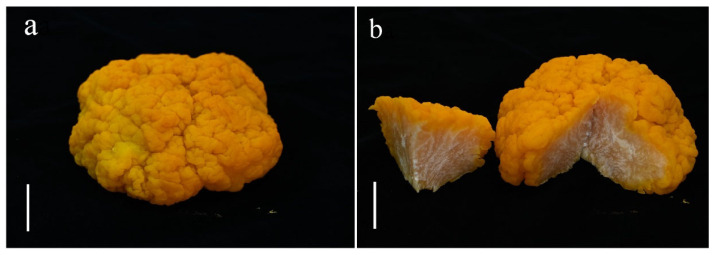
*Naematelia sinensis* (HKAS144460): (**a**) fresh basidioma; (**b**) vertical sections of basidioma. Bars = 20 mm.

**Figure 9:**
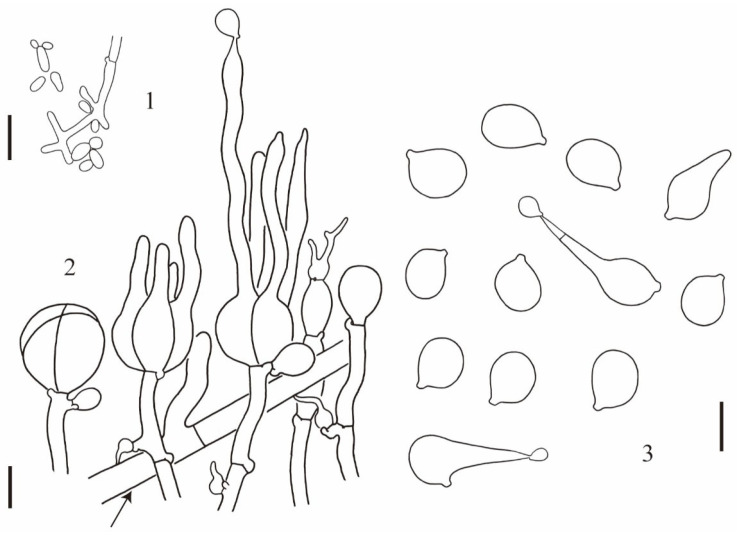
*Naematelia sinensis* (HKAS144460): (1) tramal hyphae with conidiophores and conidia; (2) hymenium with basidia at different stages of development, with haustoria attached to unclamped hypha of host (arrow); (3) basidiospores, germinating by repetition. Bars = 10 μm.

***Naematelia sinensis* Zhu L. Yang & J.Y. Tang, sp. nov. (Figure 8 and Figure 9).**

**MycoBank:** MB 856510

**Etymology:** Named for its occurrence in China.

**Type:** China, Yunnan Province, purchased from a market in Kunming, 28 July 2024, Qi Zhao JE01 (HKAS 144465, holotype).

**Diagnosis:** This species resembles *N. aurantialba* but differs from the latter in its smaller basidia and smaller basidiospores and distribution in subtropical areas at altitudes of 1800–2600 m above sea level.

**Description:** Basidioma up to 5 cm high and 5–7 cm in diameter, globose to subglobose to hemispherical, lemon yellow to yellow brown, firm and gelatinous; surface wrinkled to contorted; flesh up to 5 cm thick, dirty white to light yellow, gelatinous, with or without cavities; brownish to brown and horny when dried (Figure 8). Mature probasidia 18–24 × 17–20 μm, subglobose, longitudinally cruciate-septate, four spored; epibasidia up to 60 μm long and 4 (6) μm in diameter, nearly cylindrical. Basidiospores [80/4/4] 9.5–11 (11.5) × 8–9.5 (10.0) μm, Q = (1.05) 1.11–1.24 (1.28), Qm = 1.16 ± 0.06, subglobose to broadly ellipsoid, smooth, germinating by budding or by repetition. Conidia 3–6 × 2–3 μm, spherical, subglobose to ovoid, thin walled, colorless, produced by conidiogenous cells. Trama with parasite hyphae and host hyphae; parasite hyphae 2–5 μm wide, colorless, thin walled to slightly thick walled, with clamp connections; haustoria numerous, clamped, haustorial filaments rarely branched; host hyphae abundant in the inner part but also present up to the subhymenium of the parasite (Figure 9).

**Habitat and distribution:** Parasitizing species of *Stereum hirsutum* or its allies growing on dead wood in subtropical broad-leaved forests in the summer and autumn at altitudes of 1800–2600 m above sea level; known from Yunnan [18].

**Additional collections examined:** China, Yunnan Province, purchased from a market in Kunming, 24 August 2024, Jin-Yan Tang 115 (HKAS 144459); 24 August 2024, Jin-Yan Tang116 (HKAS 144460); 10 September 2024, Jin-Yan Tang117 (HKAS 144461); 10 September 2024, Jin-Yan Tang118 (HKAS 144462); 28 July 2024, Jin-Yan Tang119 (HKAS 144463); 24 August 2024, Jin-Yan Tang120 (HKAS 144464).

**Notes:*** Naematelia sinensis* possesses subglobose-to-hemispherical basidiomata with a yellow-to-orange-yellow brain-like surface and without internal cavities. It resembles *N. aurantialba* but differs from the latter in its smaller basidia and smaller basidiospores and its distribution in subtropical areas at altitudes of 1800–2600 m above sea level.

*Naematelia sinensis* looks like *N. encephaloidea* A. Thomas & T.K.A. Kumar, originally described from India. However, the latter species has much smaller basidia, measuring 11–16 × 10–13 μm, and significantly smaller basidiospores, measuring 6.2–9 × 5–7 μm [12].

## 4. Discussion

Southwestern China is extraordinarily rich in fungi, and the Eastern Himalayas and Hengduan Mountain region is one of the three eminent centers of higher fungi in China [35] and one of the world’s important centers of speciation and differentiation of fungi resulting from the comprehensive impacts of the upward thrust of mountains, monsoon development, and climate fluctuations in geographic history [36]. Our molecular phylogenetic analysis revealed that the sequences of samples from southwestern China submitted to GenBank under the name “*Naematelia aurantialba*” were clustered in three different species lineages, namely *N. sinensis*, *N. aurantialba*, and *N. nodulosa* (Figure 1), indicating that there is a species complex with similar macro-morphological features in the region. Each species of the complex has, however, adapted to specific altitudes, environment conditions, and/or vegetation types.

Growing on the decayed wood of *Quercus* species, *N. aurantialba* occurs in subalpine temperate areas over 3000 m above sea level, suggesting that it may be a species with a preference for low temperatures during stages of basidioma development, like *Flammulina filiformis* (Z.W. Ge et al.) P.M. Wang et al. [37]. In contrast, *N. sinensis* is mainly distributed in subtropical broad-leaved forests at altitudes of 1800–2600 m. The optimal temperature for mycelium growth is 20–25 °C, while for fruiting, it is 18–22 °C [18]. This species has commercially been cultivated at room temperature in middle and northern subtropical areas in Yunnan without any cooling device. Because species of *Naematelia* are mycoparasitic [15], *N. sinensis* has been cocultured with the host *Stereum* [18], and therefore, unclamped hyphae of the host were observed in the specimens examined under a microscope (Figure 9).

This study provides new insights into the species diversity of *Naematelia* and taxonomic information for future strain improvements, cultivation practices, and conservation strategies for these valuable fungi. For the convenience of recognizing the species, a key to them is furnished next.

**A key to the species of *Naematelia aurantialba* complex in southwestern China**

1 Basidiomata occur in subtropical forests at altitudes of 1800–2600 m above sea level………………………………………………………………………………… 2
1′ Basidiomata occur in subalpine temperate forests at altitudes of 3000–3800 m above sea level………………………………………………………………… ***N. aurantialba***
2 Surface of basidioma knob-like, trama with small cavities, basidia two spored, conidia numerous and everywhere…………………………………………………***N. nodulosa***
2′ Surface of basidioma cerebriform, trama with or without small cavities, basidia four spored, conidia abundant to numerous……………………………………………… 3
3 Surface of basidioma yellow to orange yellow, basidia not stalked, basidiospores 9.5–11 μm long, with Q = 1.11–1.24……………………………….….…………………***N. sinensis***
3′ Surface of basidioma dull ochraceous, basidia long stalked (stalks up to 40 μm long), basidiospores 10.5–12.5 μm long, with Q = 1.18–1.38……………………… ***N. pedicellata***

## Figures and Tables

**Table 1:** DNA sequences used in this study.

Current Name	Voucher	Country (Province) Where Material Collected	ITS	nrLSU
*Dimennazyma* *. cisti-albidi*	CBS 10049	-	KF036589	KY107630
*D* *. cisti-albidi*	WOct07D (2)-Y15	-	NR_144841	GQ244505
*Naematelia aurantia*	WA0000072740	Poland	MT229987	-
*N. aurantia*	LE 253851	Russia	KP986510	KP986543
*N. aurantia*	CBS 6965	Netherlands	AF444315	AF189842
“*N. aurantialba*”	9102	China	DQ404321	EF010939
“*N. aurantialba*”	9901	China	DQ400104	EF010937
“*N. aurantialba*”	NX-20	China	OQ629799	-
“*N. aurantialba*”	Dai 23432	China	OL614834	OL616185
“*N. aurantialba*”	WJ-2	China	PP917728	-
“*N. aurantialba*”	NA	China	PQ084746	-
“*N. aurantialba*”	WJ-1	China	PP917727	-
“*N. aurantialba*”	down1	China	PQ084585	-
“*N. aurantialba*”	Dong1651b	China	PQ097671	--
“*N. aurantialba*”	Dong1633	China	PQ097670	-
“*N. aurantialba*”	1554	China	PP859876	-
“*N. aurantialba*”	Dai20202	China	OL655278	-
“*N. aurantialba*”	ACCC 50219	China	AY866425	-
*N. aurantialba*	HKAS19954 *	China	PQ650944	PQ654928
*N. aurantialba*	HKAS18922	China	PQ650943	PQ654927
*N. aurantialba*	HKAS45544	China	PQ650946	PQ654930
*N. aurantialba*	HKAS53591	China	PQ650945	PQ654929
*N. aurantialba*	HKAS89568	China	PQ650948	PQ654932
*N. aurantialba*	HKAS90938	China	PQ650947	PQ654931
*N. encephala*	yHKS553	USA	OK051297	-
*N. encephala*	FBCC1144	Finland	EU673082	-
*N. encephala*	FBCC1145	Finland	EU673083	-
*N. encephala*	LE 253857	Russia	KP986506	KP986540
*N. encephala*	LE 253860	Russia	-	KP986564
*N. encephala*	CBS 8220	-	KY104315	
*N. encephala*	CBS 8218	-	KY104314	
“*N. encephaloidea*”	ZGCAT325	India	OQ621803	-
“*N. encephaloidea*”	ZGCAT329	India	OQ621704	-
*N. encephaloidea*	ZGCAT326 *	India	OQ621795	OQ621978
*N. encephaloidea*	ZGCAT327	India	OQ621748	-
*N. microspora*	BPI702328 *	South Africa	AF042435	AF042253
*N. nodulosa*	HKAS141719 *	China	PQ650958	PQ654942
*N. nodulosa*	HKAS141591	China	PQ650959	PQ654943
*N. nodulosa*	HKAS141353	China	PQ650957	PQ654941
*N. nodulosa*	HKAS141704	China	PQ650956	PQ654940
*N. pedicellata*	HKAS112730 (a) *	China	PQ650960	PQ654944
*N. pedicellata*	HKAS112730 (b)	China	PQ650961	PQ654945
*N. pedicellata*	HKAS112730 (c)	China	PQ650962	PQ654946
*N. sinensis*	HKAS144465 *	China	PQ650952	PQ654936
*N. sinensis*	HKAS144459	China	PQ650955	PQ654939
*N. sinensis*	HKAS144460	China	PQ650953	PQ654937
*N. sinensis*	HKAS144461	China	PQ650951	PQ654935
*N. sinensis*	HKAS144462	China	PQ650954	PQ654938
*N. sinensis*	HKAS144463	China	PQ650949	PQ654933
*N. sinensis*	HKAS144464	China	PQ650950	PQ654934

Type specimens are marked with an asterisk (*).

## Data Availability

The original contributions presented in the study are included in the article, further inquiries can be directed to the corresponding author.
